# Retrospective analysis of the accuracy of predicting the alert level of COVID-19 in 202 countries using Google Trends and machine learning

**DOI:** 10.7189/jogh.10.020511

**Published:** 2020-12

**Authors:** Yuanyuan Peng, Cuilian Li, Yibiao Rong, Xinjian Chen, Haoyu Chen

**Affiliations:** 1 School of Electronics and Information Engineering, Soochow University, Suzhou, China; 2 Joint Shantou International Eye Center, Shantou University and the Chinese University of Hong Kong, Shantou, China; 3 College of Engineering, Shantou University, Shantou, China

## Abstract

**Background:**

Internet search engine data, such as Google Trends, was shown to be correlated with the incidence of COVID-19, but only in several countries. We aim to develop a model from a small number of countries to predict the epidemic alert level in all the countries worldwide.

**Methods:**

The “interest over time” and “interest by region” Google Trends data of Coronavirus, pneumonia, and six COVID symptom-related terms were searched. The daily incidence of COVID-19 from 10 January to 23 April 2020 of 202 countries was retrieved from the World Health Organization. Three alert levels were defined. Ten weeks' data from 20 countries were used for training with machine learning algorithms. The features were selected according to the correlation and importance. The model was then tested on 2830 samples of 202 countries.

**Results:**

Our model performed well in 154 (76.2%) countries, of which each had no more than four misclassified samples. In these 154 countries, the accuracy was 0.8133, and the kappa coefficient was 0.6828. While in all 202 countries, the accuracy was 0.7527, and the kappa coefficient was 0.5841. The proposed algorithm based on Random Forest Classification and nine features performed better compared to other machine learning methods and the models with different numbers of features.

**Conclusions:**

Our result suggested that the model developed from 20 countries with Google Trends data and Random Forest Classification can be applied to predict the epidemic alert levels of most countries worldwide.

In December 2019, several cases with pneumonia of unknown cause were reported in Wuhan city, Hubei province, China [1]. It quickly spread in China and globally. Later, a novel coronavirus was identified as the cause of pneumonia by the Chinese Center for Disease Control and Prevention [2]. The disease was named as Coronavirus Disease 2019 (COVID-19) by the World Health Organization (WHO) [2]. On 30 January 2020, WHO declared COVID-19 outbreak as a Public Health Emergency of International Concern [3]. On 11 March 2020, COVID-19 was defined as a worldwide pandemic by WHO. As of 30 April 2020, this disease has been reported in more than 200 countries, with 3 090 445 confirmed cases and 217 769 deaths.

As COVID-19 is a rapidly spreading infectious disease, it is crucial to predict the outbreak in a specific country or region as early as possible, for taking sooner action to prevent its spread. Traditional surveillance systems rely on both clinical and virological data, which may lead to days or weeks reporting lag. Internet data, such as web-search engine and social media, have been applied to monitor the outbreak of infectious diseases such as influenza [4], Dengue [5], H1N1 [6], Zika [7], measles [8], Middle East respiratory syndrome [9]. Recently, it is reported that Google Trends data using search terms relative to COVID-19, such as “coronavirus”, “pneumonia”, “handwashing”, “face masks” were correlated with the officially reported number of confirmed COVID-19 cases in China [10], South Korea, Italy, Iran [11], USA [12], and other countries. Besides the correlation analysis, data mining and deep learning technique were also used to model Google Trends data and predict the incidence of COVID-19 in Iran [13].

However, the published articles only investigated the data on one or several countries. Some articles studied worldwide Google Trends data but taking the world as a whole [14]. The deep learning algorithm developed from the data of Iran was used to predict the incidence of COVID-19 in the same country only [13]. In this study, we aimed to evaluate the accuracy of our machine learning algorithm developed from the Google Trends data of 20 countries in predicting the weekly alert level of COVID-19 pandemic in all the individual countries worldwide.

## METHODS

### Data sources

We use two sets of data to train and evaluate models. The first data set is the search volume data obtained from the Google Trends service. We collected Google search volume of 16 candidate features relating to COVID-19 for 15 weeks from 3 January 2020 to 16 April 2020 in 202 countries. Eight terms were used for search as topics on Google Trends, including “Coronavirus”, “Pneumonia”, and six symptom-related terms [15], “Cough”, “Diarrhea”, “Fatigue”, “Fever”, “Nasal congestion” and “Rhinorrhea”. We selected the term “Coronavirus” instead of “Covid-19”. The reason is that at the early stage of epidemic, the public didn’t know about this novel disease very clearly in most countries, but only recognize that this disease was caused by a novel coronavirus. Meanwhile, the correlation coefficient between “Coronavirus” and daily confirmed cases was slightly higher than “COVID-19” on the whole (Table S1 in the **Online Supplementary Document**). Two types of data were retrieved. In the first one, the data of “interest over time” was defined as the search interest relative to the highest point for the specific term, region, and time interval. Values are calculated on a scale from 0 to 100, where 100 is the peak popularity for the term. The second one, the data of “interest by region” was retrieved by setting the region to “worldwide” for the given term and time. The values were calculated on a scale from 0 to 100, where 100 is the location with the most popularity as a fraction of total searches in that location. It's worth noting that we included low search volume regions for obtaining more regions’ data. The features of “interest by region” was named as “Coronavirus_RE”, “Pneumonia_RE”, “Cough_RE”, “Diarrhea_RE”, “Fatigue_RE”, “Fever_RE”, “Nasal congestion_RE” and “Rhinorrhea_RE”.

The second data set is the daily number of COVID-19 new cases each day from 10 January to 23 April 2020 in 202 countries from WHO website [16]. In this study, we defined the weekly epidemic alter of COVID-19 in a specific country into three levels. The higher the alert level, the higher the risk of an outbreak in this week. Suppose *m_d_* denotes the number of newly confirmed cases on day d within the seven days of the week. Let *x_t_* denotes the alert levels of the week t. The epidemic alert level of week t can be obtained according to the following formula:

1) If all *m_d_* = 0 (d = 1, 2, 3, 4, 5, 6, 7), *x_t_* = 1, else

2) If 0< *m_d_* <10 at least one day, *x_t_* = 2, else

3) *x_t_* = 3

**Figure 1:**
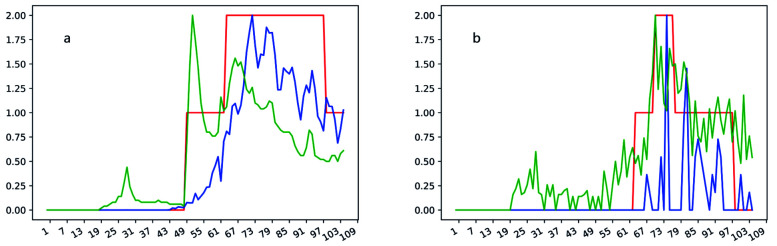
The predicted alert level (red), normalized Google Trends search volume of the topic “Coronavirus” (green), normalized daily new confirmed cases. **Panel A.** Italy. **Panel B.** United States Virgin Islands.

Based on the correlation analysis between Google search volume data and the daily new confirmed cases, we randomly selected 20 countries from the countries with strong correlation as the training set. Ten samples, which are the Google search volume data from 10 January 2020 to 19 March 2020, from each of the 20 countries were used in the training stage. Totally 200 samples, including 87 samples with alert level 1, 43 samples with alert level 2, and 70 samples with alert 3, were used for training. The remaining 2830 samples from 202 countries are used for model testing, including 2730 data for 1 to 15 weeks in the other 182 countries and the remaining 100 data in the 20 training set countries. Examples of the search volume of the term “Coronavirus” and the daily incidence of COVID-19 in Italy and US Virgin Island were shown in **Figure 1**.

### Feature engineering

Feature engineering of data are to extract the features that have a great or small influence on output results from various parameters and use these features as the basis of the training model. In the beginning, we included 16 features. Two kinds of correlation analyses were conducted in each country to select the best terms from the candidate set. The first one is the correlation between Google search volume data of each feature and daily new confirmed cases at one week behind, in which “Daily_AVG” and “Daily_MAX” were used to represent the average and maximum Spearman correlation coefficients for the 202 countries. The other one is the correlation between the average weekly Google search volume data of each feature and the weekly epidemic alert level one week behind, in which “Label_AVG” and “Label_MAX” were used to represent the average and maximum Spearman correlation coefficients in the 202 countries. The results were shown in Table 1, which showed that the top-related term was the interest over time of “Coronavirus”, while the correlation between the features of “Diarrhea”, “Fatigue”, “Nasal congestion_RE” and “Fatigue_RE” and target value was negative or weak. Therefore, we first removed the four features that are not related to the target value.

**Table 1:** Spearman correlation coefficients of 16 features with the incidence of COVID-19 at one-week lag in 202 countries*

Features	Daily_AVG	Daily_MAX	Label_AVG	Label_MAX
Coronavirus	0.59	0.88	0.78	0.93
Pneumonia	0.15	0.80	0.31	0.91
Cough	0.09	0.72	0.23	0.91
Fever	0.16	0.80	0.31	0.92
Nasal congestion	0.09	0.69	0.22	0.88
Rhinorrhea	0.16	0.76	0.35	0.92
Diarrhea	0.01	0.70	0.02	0.82
Fatigue	-0.03	0.52	-0.01	0.89
Coronavirus_RE	0.38	0.75	0.60	0.90
Pneumonia_RE	0.13	0.75	-0.16	0.77
Cough_RE	0.09	0.68	0.20	0.87
Fever_RE	0.04	0.47	0.27	0.59
Nasal congestion_RE	-0.06	0.43	-0.07	0.85
Rhinorrhea_RE	0.01	0.45	0.01	0.78
Diarrhea_RE	-0.04	0.29	0.23	0.88
Fatigue_RE	-0.12	0.27	-0.03	0.75

*Daily_AVG and Daily_MAX: the average and maximum Spearman correlation coefficient of Google search volume data of each feature and daily new confirmed cases at one week behind in 202 countries; Label_AVG and Label_MAX: the average and maximum Spearman correlation coefficient of the average weekly Google search volume data of each feature and the weekly epidemic alert level one week behind in 202 countries.

Since there is a certain correlation between the features [17-20], we used Spearman correlation coefficients to analyze the daily and weekly search volume of the 12 input features in 202 countries. We found strong correlations between “Coronavirus_RE”, “Pneumonia_RE” and “Rhinorrhea_RE” features and other features in most countries. Therefore, we further removed these three features, and finally take the remaining nine features as the input features of this study, which are “Coronavirus”, “Pneumonia”, “Cough”, “Fever”, “Nasal congestion”, “Rhinorrhea”, “Cough_RE”, “Fever_RE”, and “Diarrhea_RE”.

### Modeling and evaluation

Random Forest Classification algorithm was utilized to predict the next week's alert level based on Google search volume data for the current week related to COVID-19. Python 3.7.6 (Python Software Foundation, Beaverton, OR, USA) was used for modeling and evaluation. After training, we can use the Google search volume data of week t to predict the alert level of week t +1. To quantitatively evaluate the performance of our model, we calculated the following five evaluation metrics: accuracy (ACC), macro precision (Macro_P), macro recall (Macro_R), macro F1-score (Macro_F) and kappa-coefficient (K_Score). They are defined as:


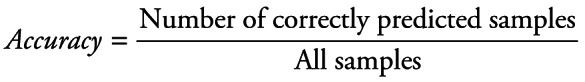
(1)


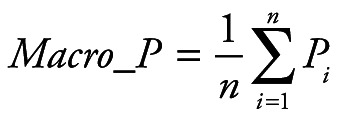
(2)


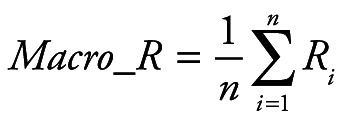
(3)


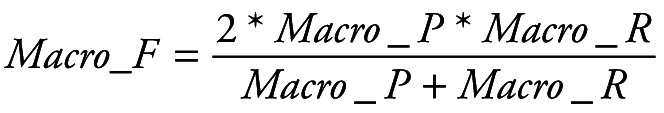
(4)


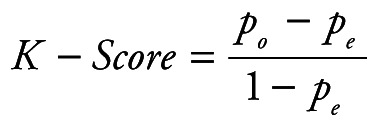
(5)

Where *P_i_* and *R_i_* represent precision and recall for the category *i*, *p_o_* equivalents to accuracy, and *p_e_* is the hypothetical probability of chance agreement, using the observed data to calculate the probabilities of each observer randomly seeing each category.

## RESULTS

We tested 2830 test sets in 202 countries and found that our model performed well in 154 (76.2%) countries, of which each country has no more than four misclassified samples. Specifically, the predictive accuracy of our model is 100% in five countries, one mistake in each of 18 countries, two mistakes in each of 43 countries, three mistakes in each of 39 countries, and four mistakes in each of 29 countries, five mistakes in each of 19 countries, six mistakes in each of 12 countries, seven mistakes in each of 6 countries, eight mistakes in each of 8 countries, 10, 11 and 12 mistakes in one country respectively. The list of the name the countries with different accuracy was shown in **Table 2**. Besides, our model predicted only two mistakes on 100 test samples of the 20 training set countries. The performance of the algorithm in different sets of data was shown in **Table 3**. Two examples were shown in **Figure 1****.**

**Table 2:** The list of countries/regions with different results

Category	No.	List of countries/regions
Training	20	Argentina, Australia, Austria, Belgium, Brazil, Finland, France, Germany, India, Indonesia, Iran (Islamic Republic of), Ireland, Italy, Peru, Poland, Puerto Rico, South Africa, Spain, Switzerland, United States of America
0 error	5	Aruba, Central African Republic, French Polynesia, Ghana, Venezuela (Bolivarian Republic of)
1 error	18	Canada, Chad, Colombia, Costa Rica, Coted Ivoire, Greece, Guadeloupe, Iceland, Kuwait, Morocco, Netherlands, Panama, Republic of Moldova, Rwanda, The United Kingdom, United States Virgin Islands, Uruguay, Uzbekistan
2 errors	43	Albania, Anguilla, Antigua and Barbuda, Bahrain, Bolivia (Plurinational State of), Botswana, Bulgaria, Burundi, Cameroon, Chile, Croatia, Cuba, Cyprus, Dominican Republic, Eritrea, Eswatini, Falkland Islands (Malvinas), French Guiana, Gambia, Grenada, Honduras, Kazakhstan, Kenya, Kyrgyzstan, Lebanon, Luxembourg, Malta, Martinique, Mauritania, Montenegro, Nigeria, Norway, Oman, Portugal, Qatar, Reuntion, Saint Kitts and Nevis, Saint Martin, Serbia, Sierra Leone, Slovakia, Slovenia, Ukraine
3 errors	39	Algeria, Armenia, Azerbaijan, Barbados, Belarus, Burkina Faso, Cayman Islands, Curacao, Czechia, Denmark, Ecuador, Egypt, El Salvador, Equatorial Guinea, Estonia, Guatemala, Guinea, Guinea-Bissau, Hungary, Jordan, Kosovo, Latvia, Lithuania, Malawi, Mali, Mauritius, Mexico, New Caledonia, Niger, Paraguay, Saint Barthelemy, Saint Vincent and the Grenadines, Senegal, Sint Maarten, Sweden, Togo, Tunisia, Turkey, Saudi Arabia
4 errors	29	Bahamas, Bangladesh, Benin, Bermuda, Bhutan, Djibouti, Gibraltar, Guernsey, Guyana, Haiti, Jersey, Liberia, Libya, Madagascar, Montserrat, Mozambique, North Macedonia, Philippines, Romania, Russian Federation, Seychelles, Somalia, South Sudan, Turks and Caicos Islands, Uganda, United Republic of Tanzania, Afghanistan, Pakistan, Sudan
5 errors	19	Bosnia and Herzegovina, Fiji, Georgia, Greenland, Guam, Isle of Man, Jamaica, Japan, Liechtenstein, Myanmar, Nepal, Papua New Guinea, San Marino, Singapore, Sri Lanka, Suriname, United Arab Emirates, Zambia, Zimbabwe
6 errors	12	Andorra, Belize, Ethiopia, Gabon, Malaysia, Mayotte, Mongolia, Nicaragua, Saint Lucia, Sao Tome and Principe, Timor-Leste, Yemen
7 errors	6	Angola, British Virgin Islands, Dominica, New Zealand, Thailand, Trinidad and Tobago
8 errors	8	Brunei Darussalam, Cambodia, Faroe Islands, Iraq, Israel, Maldives, Northern Mariana Islands (Commonwealth of the), Syrian Arab Republic
10 errors	1	China
11 errors	1	Viet Nam
12 errors	1	Laos

**Table 3:** Performance of the final model in different test data sets

Test data	ACC	M_P	M_R	M_F1	K-Score
20 training +5 no error countries	0.9886	0.9781	0.9912	0.9844	0.9803
43 countries with ≤1 error	0.9528	0.9195	0.9306	0.9248	0.9209
86 countries with ≤2 errors	0.9009	0.8633	0.8458	0.8534	0.8324
125 countries with ≤3 errors	0.8663	0.8170	0.7995	0.8058	0.7729
154 countries with ≤4 errors	0.8133	0.7489	0.7377	0.7401	0.6828
All 202 countries	0.7527	0.6724	0.6754	0.6698	0.5841

ACC – accuracy, M_P – macro precision, M_R – macro recall, M_F1 – macro F1-score, K_Score – kappa-coefficient

**Figure 2:**
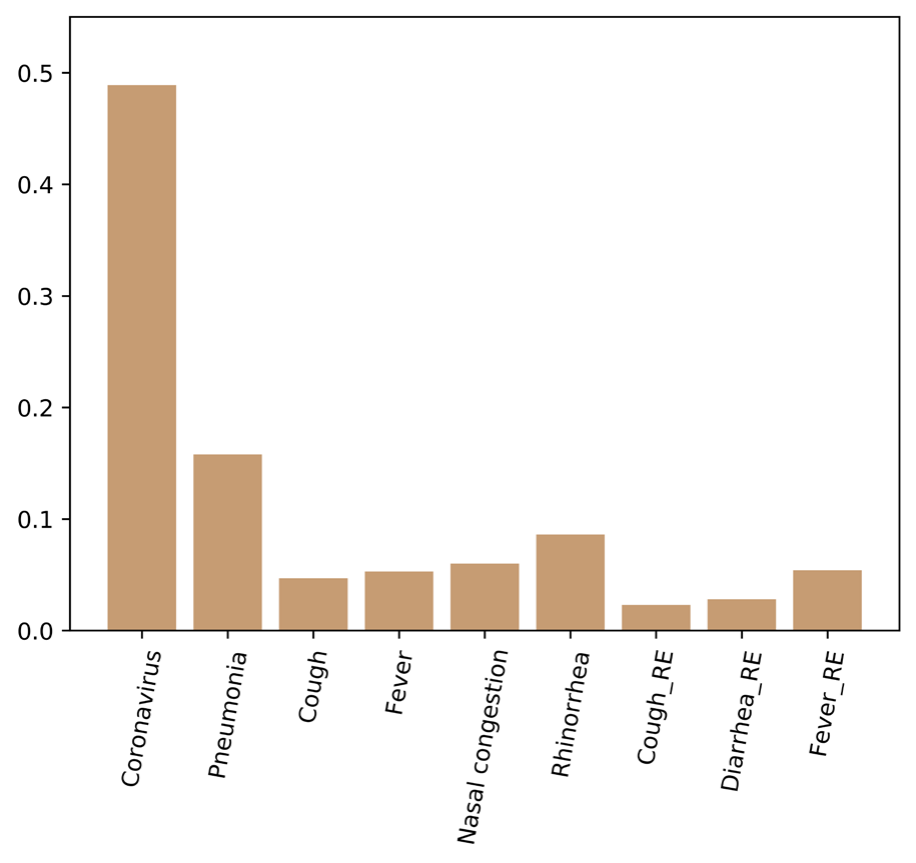
The importance of included features.

To evaluate the role of each input feature in classification, we calculated the importance score of each input feature. The higher the score, the more important the feature. As shown in **Figure 2**, the most important feature was the term “Coronavirus”, which was consistent with the results of correlation analysis.

For the real-time prediction of weekly alert level, the proposed Random Forest Classification algorithm was compared with other common machine learning classification methods: Linear Regression Classification (LRC) [21], Support Vector Machine (SVM) [22], k-Nearest Neighbor (K-NN) [23], Decision Tree Classification (DTC) [24]. **Table 4** showed the quantitative results of different methods on the test data set. As can be observed, the Random Forest Classification algorithm achieved much better results in terms of all quantitative metrics compared to other methods.

**Table 4:** Performance of different machine learning methods in 202 countries

Methods	ACC	M_P	M_R	M_F1	K_Score
Linear Regression Classification	0.6127	0.5138	0.5472	0.5048	0.3786
Support Vector Machine	0.5943	0.4963	0.5080	0.4998	0.3210
k-Nearest Neighbor	0.6799	0.5962	0.5516	0.5592	0.4156
Decision Tree Classification	0.6681	0.6027	0.6152	0.6064	0.4605
Random Forest Classification	**0.7527**	**0.6724**	**0.6754**	**0.6698**	**0.5841**

ACC – accuracy, M_P – macro precision, M_R – macro recall, M_F1 – macro F1-score, K_Score – kappa-coefficient

A series of ablation studies were conducted to validate the features included in this study. As you can see in **Figure 2**, the features of “Cough_RE” and “Diarrhea_RE” contribute little to classification. Therefore, we removed these two features and selected the other seven features as the input. Also, we trained the model with all 16 features as input. The experimental results were shown in **Table 5**. The proposed method (9 features) outperformed other methods on all metrics, including 16 features and 7 features as input. In terms of accuracy, the performance of the proposed method was 0.26% and 0.79% higher than the other two methods respectively.

**Table 5:** The result of ablation experiments in 202 countries

Model	ACC	M_P	M_R	M_F1	K_Score
7 features	0.7501	0.6705	0.6732	0.6691	0.5821
9 features	**0.7527**	**0.6724**	**0.6754**	**0.6698**	**0.5841**
16 features	0.7448	0.6570	0.6592	0.6511	0.5686

ACC – accuracy, M_P – macro precision, M_R – macro recall, M_F1 – macro F1-score, K_Score – kappa-coefficient

## DISCUSSION

In this research, the model based on the Random Forest Classification algorithm developed from 20 countries can predict the COVID-19 epidemic alert level of next week using Google search volume data. The model performed well in 154 out of 202 countries with an accuracy of 0.8133. While in a total of 202 countries, the accuracy was 0.7527.

To the best of our knowledge, the current study is the first one to demonstrate that the Google Trends data can be used in predicting disease alert level in most countries worldwide, even the model was developed from the data of only 10% of countries. Before our study, there were some articles reported the correlation of Google Trends data with the incidence of COVID-19 in some individual countries [11-13] or taking the world as a region without the information of individual countries [14]. However, there are different terms related to COVID-19. Therefore, Machine learning was used to manage the big data and develop a model to predict the incidence of COVID-19, but again, only in individual countries [13]. Before COVID-19, the data of internet search engines were also be used to predict the epidemic of infectious diseases. Google Flu Trends provided estimates of influenza activity for using the data of Google Search queries. But it only offered the prediction for 29 countries [25]. Our model has high accuracy predicting the alert level of COVID-19 in most countries. There was no mistake in five countries, one mistake in each of 18 countries, two mistakes in each of 43 countries, three mistakes in each of 39 countries, and four mistakes in each of 29 countries. That is, in more than 75% of countries, our model made no more than four mistakes in 15 predictions.

**Figure 3:**
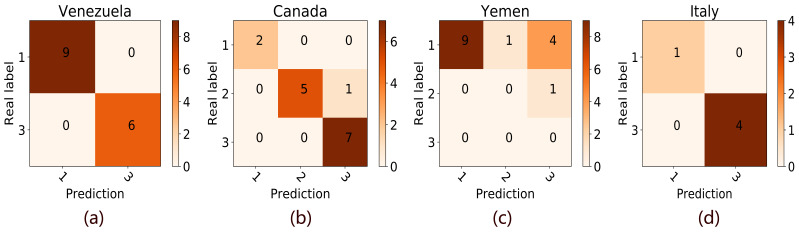
Classification confusion matrix.

We demonstrated the results of four countries (Venezuela, Canada, Yemen, and Italy) randomly selected from the countries with zero mistakes, no more than 4 mistakes, more than 4 mistakes, and the training data set in **Figure 3**. As can be seen from **Figure 3**, our model tends to mispredict a low alert level into a high level in Yemen. This phenomenon is also prevalent in the forecasts of many countries. The false-positive mistake may because that COVID-19 is a global epidemic, which has attracted full attention in the world. Therefore, the occurrence of COVID-19 in some countries will increase the awareness of other countries, especially the countries with a close relationship with the outbreak countries, and result in a large amount of Google search volume for terms related to COVID-19. On the other hand, our model also false-negatively predicted a high alert level into a low alert level in a few countries. The possible reason is that the Google search engine is not the mainstream search engine in these countries. Further studies were needed to correct the misprediction in these countries.

There are also some limitations in this study. Our model is based on data from the early stages of the epidemic, when most countries were unprepared for the outbreak. As the pandemic progressing, the public and governments have already known what to do with COVID-19. Therefore, our model may not be applicable at a later stage of the pandemic. Another point is that the COVID-19 outbreak has occurred in most countries, and it is not necessary to predict the outbreak. However, our model cannot only be used to predict the outbreak, but also to predict the recovery (Figure 1). Therefore, it still has merits even in the time of the global pandemic. Furthermore, our methods provide a new way of predicting global novel infectious diseases outbreak and can be used in other epidemics.

## CONCLUSIONS

We used a machine-learning algorithm to model the data of Google Trends data from 20 countries. The results achieved real-time epidemic alert level prediction of COVID-19 one week behind in 154 countries at the early stages of epidemic.

## Additional material

Online Supplementary Document
