# Supplementary material for: Retrospective analysis of the accuracy of predicting the alert level of COVID-19 in 202 countries using Google Trends and machine learning
Source: J Glob Health. 2020 Sep 23;10(2):020511. doi: 10.7189/jogh.10.020511 (PMC7567446; doi:10.7189/jogh.10.020511)
Supplement: Online Supplementary Document [file jogh-10-020511-s001.pdf]

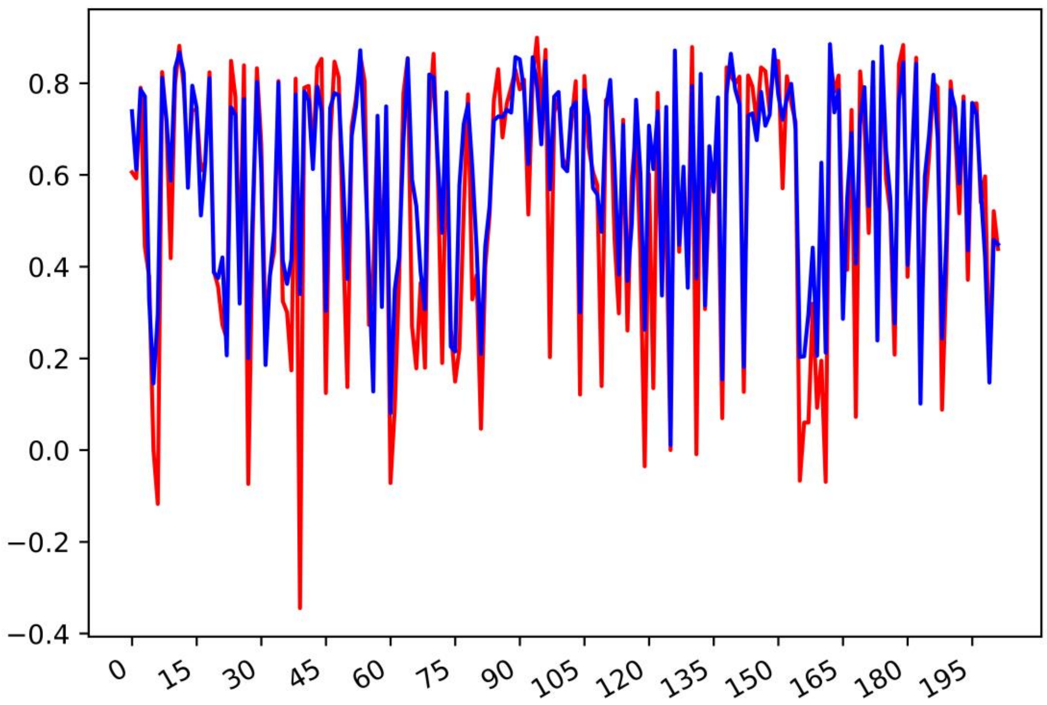

Figure S1. Correlation coefficients between “Coronavirus”/ “COVID-19” and daily confirmed cases in 202 countries. The blue line is “Coronavirus” and the red line is “COVID-19”.
